# Association between hyperuricemia and metabolic syndrome: A cross-sectional study in Tibetan adults on the Tibetan plateau

**DOI:** 10.3389/fendo.2022.964872

**Published:** 2022-10-20

**Authors:** Shaoli Yao, Yao Zhou, Li Xu, Qi Zhang, Shimin Bao, Huiru Feng, Weihong Ge

**Affiliations:** Cadre Medical Department, Hospital of Chengdu Office of People’s Government of Tibet Autonomous Region, Chengdu, Sichuan, China

**Keywords:** hyperuricemia, metabolic syndrome, Tibetan plateau, components of metabolic syndrome, Tibetan adults

## Abstract

**Purpose:**

This study aimed to assess the relationship of serum uric acid with metabolic syndrome and its components in Tibetan adults on the Tibetan plateau.

**Methods:**

A total of 307 participants were enrolled in this study and biochemical parameters including serum uric acid, fasting plasma glucose, white blood cell, lymphocyte count, mononuclear cells, alanine aminotransferase, aspartate aminotransferase, creatinine, and lipid profile were analyzed using standard methods. The IDF criteria were applied to define metabolic syndrome. The association of serum uric acid with metabolic syndrome and its components was evaluated by multivariable logistic regression models.

**Results:**

The overall prevalence of metabolic syndrome was 17.3% (53/307) with 19.6% (31/158) in females and 14.8% (22/149) in male participants. The prevalence of hyperuricemia was 40.7% (125/307) with significant differences between the male (53.7%,80/149) and female (28.5%,45/158) groups. In regression analysis, we observed that the risk of MetS was higher in participants in the hyperuricemia group (adjusted OR, 4.01; 95% CI, 2.02~7.99) compared with those in the normouricemia group. After adjusting for all confounding factors, a 9% higher risk of MetS could be shown in participants with SUA increased per 10umol/L (adjusted OR, 1.09; 95% CI, 1.04~1.14). These relationships were not affected by sex or age (*p >*0.05). After adjusting for the confounding factors, hyperuricemia is positively associated with abdominal obesity (adjusted OR, 2.53; 95% CI, 1.41~4.53), elevated blood pressure (adjusted OR, 2.61; 95% CI, 1.37~4.97), and elevated triglycerides(adjusted OR, 2.47; 95% CI, 1.09~5.57).

**Conclusions:**

In our study, hyperuricemia is significantly associated with the prevalence of metabolic syndrome and part of its components, and these relationships are not affected by sex or age. Given the high prevalence of MetS and hyperuricemia among Tibetan adults, more studies are required to explore the role of SUA in the pathogenesis of MetS.

## Introduction

Metabolic syndrome (MetS) is a group of interrelated metabolic abnormalities such as elevated blood pressure, hyperglycemia, central obesity, insulin resistance, and dyslipidemia (1). Individuals with MetS are more susceptible to cardiovascular diseases (CVD), type 2 diabetes mellitus (T_2_DM), and certain aggressive cancers, which are the major causes of mortality worldwide (2–5). The prevalence of MetS was different worldwide due to the geographic location and criteria used, varying from 10–40% (6), and the prevalence of MetS is increasing at an alarming rate both in developed and developing countries. A 2002 survey on the nutrition and health of the Chinese population indicated that 2.6% of Tibetans had MetS after adjusting for age and sex (7). 8.2% of Tibetans in Tibet’s Lhasa area were found to have MetS among Tibetan farmers and herders (8).

Serum uric acid (SUA) is the catabolic product of exogenous dietetic compounds and endogenous purines (9). Recent epidemiological studies indicate that 8.4–25% of the general Chinese population is diagnosed with hyperuricemia (10). Elevated SUA was found to have an adverse impact on mortality in the general adult population, particularly in older (>50 years) women (11). The findings of population studies conducted in different parts of the world are consistent with a strong relationship between elevated SUA levels and the presence of MetS (12–16). Some studies found that the risk of hyperuricemia and MetS varied by age and sex, while others have not found this phenomenon (12, 13, 15, 17). Studies in Peru and Saudi Arabia both suggest that the prevalence of MetS and obesity is higher at high altitudes than at sea levels (18, 19). Tibetans are one of the world’s largest and oldest high-altitude indigenous peoples. Tibet Autonomous Region, located in the western section of China on the Himalayan plateau, is home to around 3 million Tibetans (8). However, information is limited regarding the relationship of SUA with MetS in Tibetan adults. Therefore, given the increased prevalence of MetS in the Tibetan population, this cross-sectional study aimed to investigate the relationship of SUA with MetS and its components in general adults. This study also aimed to assess whether the risk of hyperuricemia and MetS varied by age and sex in this population.

## Materials and methods

### Study population 

**Figure 1:**
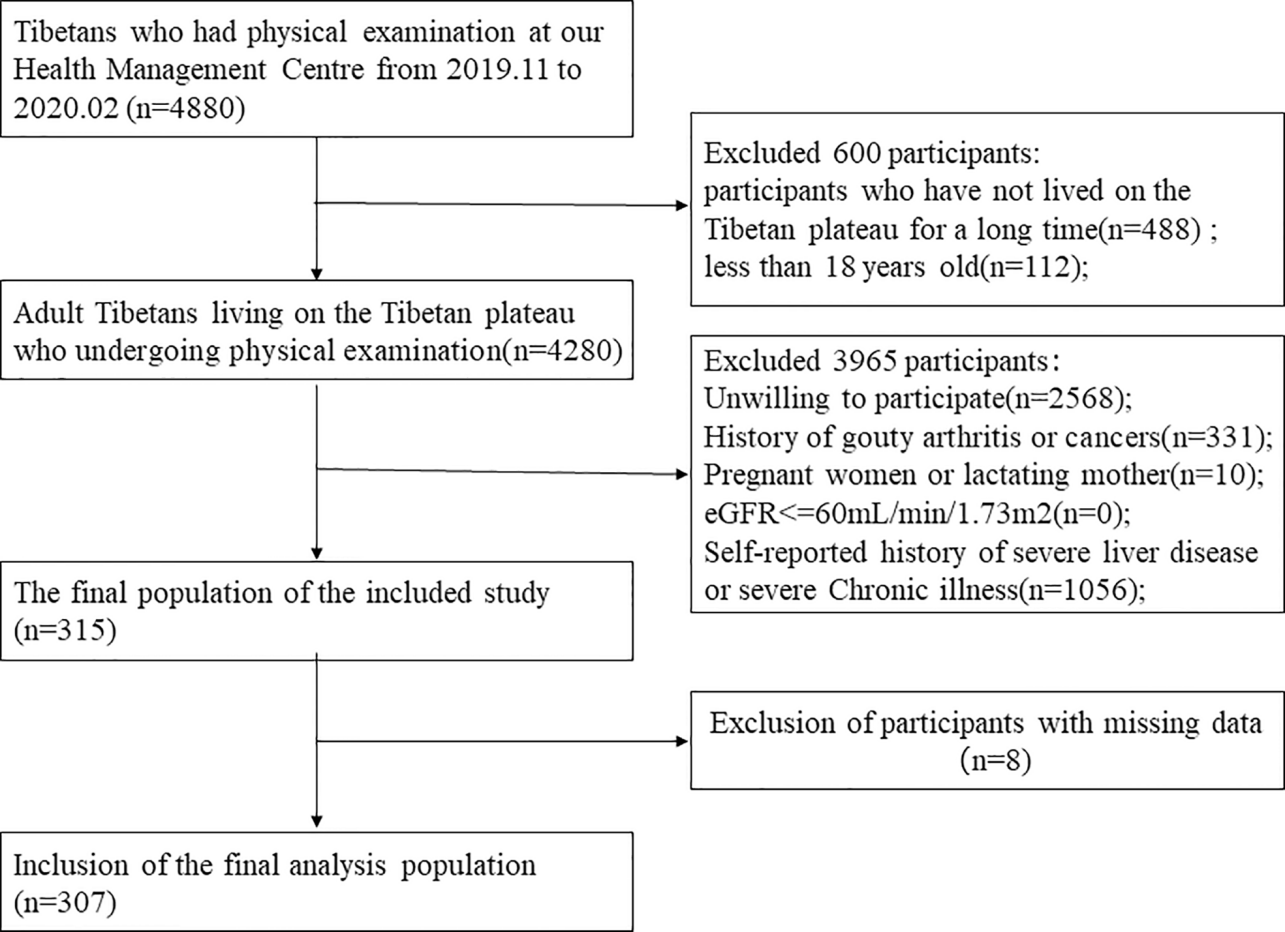
The association between Hyperuricemia and Metabolic syndrome. The Flow chart of the study.

This study was a cross-sectional design conducted between November 2019 and February 2020 at the Department of Health Management Centre of the Hospital of Chengdu Office of People’s Government of Tibetan Autonomous Region. The inclusion criteria were: aged above 18 years, born and living in the Tibetan plateau, free from gouty arthritis and cancers, free from severe chronic illness, and willing to participate. Exclusion criteria were: pregnant women, lactating mothers, subjects with missing or erroneous information on covariates, estimation of glomerular filtration rate(eGFR)<=60 mL/min/1.73m^2^, and self-reported history of severe liver disease. The detailed flow chart about participant recruitment was shown in **Figure 1**. The Hospital of Chengdu Office of People’s Government of Tibetan Autonomous Region’s ethics committee approved the study. Signed informed consent was obtained from all participants at study enrollment. Statement all methods were performed in accordance with the relevant guidelines and regulations.

### General data collection 

A standard questionnaire was used to collect demographic and lifestyle information from the participants. Individual anthropometric data such as age, sex, weight, and height were recorded in the hospital’s electronic medical record system. Briefly, Anthropometric data were collected during the visit for the physical examination of the participants. Weight and height were measured according to the recommendations of the World Health Organization, with an accuracy to the nearest 0.1 kg and 0.1 cm respectively, with the participants in lightweight clothing without shoes. BMI was calculated as weight/height^2^ (kg/m^2^). Blood pressure was measured twice using a digital sphygmomanometer according to the standard protocol in a sitting resting position after at least 5 min of rest, and the average is calculated. Waist circumference (WC) was measured using general tape that was placed midway between the lowest border of the ribs and the iliac crest. The smoking status was determined by “Current or former smoker”, and the alcohol consumption was assessed by “Current or former alcohol drinker”.

### Blood sample collection and laboratory measurements

Blood samples from participants who underwent overnight fasting were collected in the morning and analyzed within an hour in the hospital. The hepatic parameters include alanine aminotransferase (ALT), aspartate aminotransferase (AST), albumin, the renal parameters including creatinine (Cr), the lipid parameters including total cholesterol (TC), triglyceride (TG), high-density lipoprotein cholesterol (HDL-C), low-density lipoprotein cholesterol (LDL-C), Fasting plasma glucose (FPG), and SUA were measured by an automatic biochemical analyzer (HITACHI 7180). The hematological parameters including white cell count (WBC), lymphocyte count (LYMH), and mononuclear cells were detected by automatic flow cytometry. The eGFR is calculated by the following formula (20):


eGFR=175*SCr^(−1.234)*Age^(−0.179)*0.79[iffemale]

The diagnostic kits for hematological parameters were purchased from Shenzhen Myriad Biomedical Electronics Company and diagnostic kits for biochemical parameters were purchased from Meikang Biotechnology Company for analysis of the above clinical parameters. The measurements were carried out according to the standard manufacturer’s protocols provided within the kit. The precision of the measurements was maintained regularly by method standard calibration.

### Diagnostic criteria

In the present study, hyperuricemia was defined as SUA concentration >420 µmol/L in men or >360 µmol/L in women (21). MetS was defined using the International Diabetes Federation (IDF) criteria (22), that is, the presence of abdominal obesity (WC > 90cm in men, and > 80cm in women), plus any two of the four additional risk factors: elevated blood pressure>=130/>=85mmHg or antihypertensive therapy, elevated fasting glucose (fasting plasma glucose >=100mg/dL (5.6 mmol/L) or history of diabetes mellitus), elevated triglycerides (triglycerides>=150mg/dL (1.7 mmol/L) or treatment), or reduced HDL-C (HDL cholesterol in men< 40mg/dL (1.03 mmol/L), and in women< 50mg/dL (1.29 mmol/L) or treatment).

### Statistical analysis

Descriptive analysis was applied to all participants. Categorical data were presented as a number (percentages), while continuous data were presented as the mean ± standard deviation or median (interquartile range), as appropriate. The differences in categorical variables between the two groups were detected by the Chi-square test or Fisher exact test as appropriate. The student *t*-test or rank-sum test was applied to assess continuous variables between the two groups, as appropriate. Multivariable logistic regression analyses (odds ratios [OR], confidence interval [CI]) were performed to assess the independent association between SUA and MetS prevalence, and between SUA and MetS components. We applied three models in the regression analysis. Multivariable models were adjusted as follows: model1 was not adjusted; model2 was adjusted for age, and sex; model3 was adjusted for age, sex, WBC, albumin, and TC. To verify the stability of our results, we performed a multivariable logistic regression analysis after diagnosing MetS according to the NCEP-ATP III diagnostic criteria (23). Subgroup analyses were conducted using stratified logistic regression models. Interaction across subgroups was tested using the likelihood ratio test. All the analyses were performed with the statistical software packages R (http://www.R-project.org, The R Foundation) and Free Statistics software version 1.5 (24). A two-tailed test was performed, and a *P<* 0.05 was considered statistically significant in our study.

## Results

### Baseline characteristics of the participants in the normouricemia and hyperuricemia group

In total, 307 consecutive general adults (aged ≥ 18 years, 149 male and 158 female) were enrolled from Tibetans undergoing medical examination at the Health Management Centre. The baseline characteristics of the participants in the normouricemia and hyperuricemia groups are presented in **Table 1**. Among the participants, 53(17.3%) subjects were diagnosed with MetS according to the diagnostic criteria. There were significant differences in the mean or median of WC, BMI, SBP, DBP, FPG, TG, TC, ALT, and AST (*p* < 0.05) for all cases) between the normouricemia and hyperuricemia groups.

**Table 1:** Baseline characteristics of the study population.

Characteristics	Total (n = 307)	Serum uric acid level		*p* value
		Normouricemia (n = 182)	Hyperuricemia (n = 125)	
Altitude, meter	3739.2 ± 501.4	3721.8 ± 490.4	3764.5 ± 518.0	0.465
Age, year	43.0 ± 12.2	41.9 ± 11.9	44.5 ± 12.7	0.064
SBP, mmHg	116.8 ± 16.3	113.7 ± 14.5	121.3 ± 17.7	< 0.001
DBP, mmHg	71.1 ± 11.1	68.8 ± 10.6	74.5 ± 11.0	< 0.001
WC, cm	85.4 ± 11.0	82.2 ± 10.1	90.0 ± 10.6	< 0.001
TG, mmol/L	1.3 ± 0.6	1.1 ± 0.5	1.5 ± 0.6	< 0.001
HDL-C, mmol/L	1.1 ± 0.2	1.1 ± 0.2	1.0 ± 0.2	0.014
FPG, mmol/L	4.9 ± 1.1	4.7 ± 0.7	5.1 ± 1.4	0.005
BMI, kg/m2	25.1 ± 3.9	24.3 ± 3.5	26.4 ± 4.2	< 0.001
WBC, × 10^9^/L	5.6 ± 1.4	5.5 ± 1.5	5.6 ± 1.3	0.634
Cholesterol, mmol/L	4.6 ± 0.9	4.5 ± 0.9	4.8 ± 0.9	0.02
LDL-C, mmol/L	3.0 ± 0.7	2.9 ± 0.7	3.1 ± 0.7	0.118
eGFR, ml/(min*1.73m^2^)	114.0 ± 20.7	115.9 ± 21.8	111.3 ± 18.7	0.052
Mononuclear cells, × 10^9^/L	0.4 (0.3, 0.5)	0.4 (0.3, 0.5)	0.4 (0.3, 0.5)	0.123
LYMPH, × 109/L	1.8 ± 0.5	1.7 ± 0.5	1.8 ± 0.6	0.052
ALT, U/L	29.0 (20.0, 45.0)	26.0 (18.0, 39.0)	36.0 (24.0, 49.0)	< 0.001
AST, U/L	21.0 (17.0, 27.5)	20.0 (16.0, 27.0)	23.0 (19.0, 28.0)	0.015
Albumin, g/L	43.6 ± 3.5	43.4 ± 3.4	43.9 ± 3.6	0.271
Sex, n (%)				< 0.001
male	149 (48.5)	69 (37.9)	80 (64)	
female	158 (51.5)	113 (62.1)	45 (36)	
Alcohol consumption, n (%)				0.003
No	138 (45.0)	95 (52.2)	43 (34.4)	
Yes	169 (55.0)	87 (47.8)	82 (65.6)	
Smoke, n (%)				0.109
No	218 (71.0)	136 (74.7)	82 (65.6)	
Yes	89 (29.0)	46 (25.3)	43 (34.4)	
T2DM, n (%)				0.125
No	300 (97.7)	180 (98.9)	120 (96)	
Yes	7 (2.3)	2 (1.1)	5 (4)	
Hypertensive, n (%)				0.021
No	268 (87.3)	166 (91.2)	102 (81.6)	
Yes	39 (12.7)	16 (8.8)	23 (18.4)	
Educationlevel4, n (%)				0.649
Primary school or less	47 (15.3)	24 (13.2)	23 (18.4)	
Secondary school	30 (9.8)	19 (10.4)	11 (8.8)	
Intermediate	91 (29.6)	55 (30.2)	36 (28.8)	
University or college	139 (45.3)	84 (46.2)	55 (44)	
Dietary habits, n (%)				0.371
Red meat+ butter tea	77 (25.1)	42 (23.1)	35 (28)	
Vegan	3 (1.0)	1 (0.5)	2 (1.6)	
Vegetables + white meat + rice	227 (73.9)	139 (76.4)	88 (70.4)	
Occupation, n (%)				0.523
Farmer and herdsman	20 (6.5)	10 (5.5)	10 (8)	
Other jobs	287 (93.5)	172 (94.5)	115 (92)	

Data are presented as the Mean (SD) or Median (IQR: Q1, Q3) for continuous variables and percentage for categorical variables.

SBP, Systolic blood pressure; DBP, Diastolic blood pressure; WC, Waist Circumference; TG, Triglycerides; LDL-C, Low density lipoprotein cholesterol; HDL-C, High density lipoprotein cholesterol; FPG, Fasting plasma glucose; BMI, Body Mass Index; WBC, white blood cell; AST, aspartate aminotransferase; ALT, alanine aminotransferase.

### Prevalence of MetS and its components in sex and hyperuricemia group

The prevalence of MetS and its components are presented in **Table 2**. Overall, the prevalence of MetS was 17.3% with14.8% in males and 19.6% in female subjects. The MetS components such as abdominal obesity and reduced HDL-C were significantly higher in females than in the male subjects (*p *<0.01 for all cases), but elevated triglyceride and elevated blood pressure were significantly higher in males than in the female subjects. The prevalence of hyperuricemia was 40.7% with significant differences between the male (53.7%) and female (28.5%) groups (*p*<0.001). In addition to reduced HDL-C, other components of MetS were significantly higher in subjects in the hyperuricemia group compared to the subjects in the normouricemia group (*p* < 0.05 for all cases).

**Table 2:** Prevalence of MetS and its components in sex and hyperuricemia group.

Parameters	Total (n = 307)	Sex			Hyperuricemia		
		Male(n = 149)	Female(n = 158)	*p* value	Normouricemia(n = 182)	Hyperuricemia(n = 125)	*p* value
Mets, n (%)	53 (17.3)	22 (14.8)	31 (19.6)	0.330	18 (9.9)	35 (28)	< 0.001
Abdominal obesity, n (%)	142 (46.3)	53 (35.6)	89 (56.3)	< 0.001	70 (38.5)	72 (57.6)	0.001
Elevated triglycerides, n (%)	57 (18.6)	42 (28.2)	15 (9.5)	< 0.001	20 (11)	37 (29.6)	< 0.001
Reduced HDL-C, n (%)	201 (65.5)	73 (49)	128 (81)	< 0.001	122 (67)	79 (63.2)	0.567
Elevated blood pressure, n (%)	70 (22.8)	44 (29.5)	26 (16.5)	0.010	24 (13.2)	46 (36.8)	< 0.001
Elevated fasting glucose, n (%)	23 (7.5)	11 (7.4)	12 (7.6)	1	8 (4.4)	15 (12)	0.023
Hyperuricemia, n (%)	125 (40.7)	80 (53.7)	45 (28.5)	< 0.001	–	–	–

Mets, Metabolic syndrome; HDL-C, High-density lipoprotein cholesterol.

### Association of SUA with the prevalence of MetS and its components

Multivariable logistic regression was performed to assess the relationship between SUA and MetS. The detailed results are presented in **Table 3**. In regression analysis, a positive association between SUA and MetS was observed for both SUA as a continuous (SUA serum levels scaled to10 µmol/L increments) and categorical variable. In our study population, we also found statistically significant differences in alcohol consumption between the hyperuricemia group and the normouricemia group(p<0.05). We performed a sensitivity analysis to adjust the confluence of covariates, such as eGFR, LYMPH, alcohol consumption, family history of cardiovascular diseases, family history of hypertensive, and family history of type 2 diabetes mellitus, and the results remained stable. After adjusting for all confounding factors, we observed that the risk of MetS was higher in participants in the hyperuricemia group (adjusted OR, 4.01; 95% CI, 2.02~7.99) compared with those in normouricemia group, and a 9% higher risk of MetS could be shown in participants with SUA increased per 10umol/L (adjusted OR, 1.09; 95% CI, 1.04~1.14). We further assessed the relationship of hyperuricemia with the individual components of MetS (**Table 4**). After adjustment for confounding factors, a positive association was observed between hyperuricemia and part of MetS components. Such as abdominal obesity (OR [95%CI], 2.53 [1.41~4.53]), elevated blood pressure (2.61 [1.37~4.97]), and elevated TG (2.47 [1.09~5.57]) but not elevated fasting glucose (1.82 [0.64~5.15]) and reduced HDL-C (0.89 [0.47~1.67]).

**Table 3:** Multivariable logistic regression analysis of the association between serum uric acid and metabolic syndrome.

Variables	Model 1		Model 2		Model 3		
	OR (95%CI	*p* value	OR (95%CI)	*p* value	OR (95%CI)		*p* value
Uric acid per10 umol/L	1.04 (1.01~1.08)	<0.001	1.09 (1.04~1.14)	<0.001	1.09 (1.04~1.14)		<0.001
Subgroups							
Normouricemia	reference		reference		reference		
Hyperuricemia	3.52 (1.88~6.57)	<0.001	3.87 (1.99~7.51)	<0.001	4.01 (2.02~7.99)	<0.001	

Model 1: no adjusted;

Model 2: adjusted for age, sex;

Model 3: adjusted for age, sex, white blood cell, Albumin, Cholesterol;

OR, odds ratios; CI, confidence interval.

**Table 4:** Multivariable logistic regression analysis of the association between hyperuricemia and metabolic syndrome components.

Variables	Model 1		Model 2		Model 3	
	OR (95CI)	*p* value	OR (95CI)	*p* value	OR (95CI)	*p* value
Obesity	2.17 (1.37~3.46)	0.001	5.32 (2.48~11.42)	<0.001	2.53 (1.41~4.53)	0.002^a^
Elevated BP	3.83 (2.18~6.73)	<0.001	3.27 (1.80~5.96)	<0.001	2.61 (1.37~4.97)	0.003^b^
Elevated FPG	2.97 (1.22~7.23)	0.017	2.91 (1.16~7.32)	0.023	1.82 (0.64~5.15)	0.258^c^
Elevated TG	3.41 (1.86~6.22)	<0.001	2.66 (1.43~4.97)	0.002	2.47 (1.09~5.57)	0.030^d^
Reduced HDL-C	0.84 (0.52~1.36)	0.488	1.3 (0.77~2.2)	0.333	0.89 (0.47~1.67)	0.707^e^

Model 1:no adjusted;

Model 2: adjusted for age, sex;

Model 3: adjusted for age, sex, Cholesterol, Albumin, white blood cell

a: model 3 plus Systolic blood pressure, diastolic blood pressure, fasting plasma glucose, triglycerides, high-density lipoprotein cholesterol

b: model 3 plus Fasting plasma glucose, triglycerides, high-density lipoprotein cholesterol, waist circumference;

c: model 3 plus Systolic blood pressure, diastolic blood pressure, triglycerides, high-density lipoprotein cholesterol, waist circumference;

d: model 3 plus Systolic blood pressure, diastolic blood pressure, fasting plasma glucose, high-density lipoprotein cholesterol, waist circumference;

e: model 3 plus Systolic blood pressure, diastolic blood pressure, fasting plasma glucose, triglycerides, waist circumference;

BP, blood pressure; FPG, Fasting plasma glucose; TG, Triglycerides; HDL-C, High-density lipoprotein cholesterol; OR, odds ratios; CI, confidence interval.

### Sensitivity analysis

After diagnosing Mets using the NCEP-ATP III diagnostic criteria (23), we conducted a multivariable logistic regression analysis to ensure the stability of our findings, and the results still showed a positive correlation between SUA and Mets (**Supplementary Tables S1**). We conducted the multivariable regression model with two additional models to demonstrate the stability of the results, and the results still indicated that SUA was strongly associated with the prevalence of MetS (**Supplementary Tables S2**). Stratification analysis and interaction analysis were further performed to explore whether the positive association between hyperuricemia and MetS was influenced by sex, age, or altitude (**Supplementary Tables S3**). Interaction analysis showed that both sex, age, and altitude did not significantly interfere with the positive association of hyperuricemia and MetS (all *p* > 0.05).

## Discussion

### The main result

In this retrospective study among the general population who underwent routine physical examination, we discovered that hyperuricemia as well as the increase of SUA level was positively associated with the presence of MetS. Among the components of MetS, hyperuricemia is only positively associated with elevated blood pressure, elevated TG, and abdominal obesity. Although, the association of SUA with MetS has been studied in different disease states and different ethnic groups (12, 13, 17, 25–29), however, limited studies have documented the information regarding the link of SUA with MetS in Tibetan adults. In this study, we report a positive association of SUA with MetS and its components in general adults in Tibet.

### SUA was positively correlated with the presence of MetS

Our findings indicated that hyperuricemia is positively associated with MetS after adjusting for other covariates, and the association did not differ by sex and age. This finding was consistent with previous studies (12, 14, 17, 26, 27, 30). However, some studies have found that the association between SUA and MetS varies by sex or age. Wen-Ko Chiou et al. suggested that SUA and the occurrence of MetS rose with increasing age in females, however, SUA values did not vary with age in males (13). A prospective cohort study in China found that hyperuricemia was a significant independent risk determinant for MetS in women (15). We analyzed these studies that are inconsistent with our results, and we speculate that the reasons for the different results may be caused by the following factors: (1) The research population is different. These studies, which were inconsistent with our findings, were targeted at Chinese residents of Taiwan, and Chinese who were free of MetS at baseline recruitment;(2) Compared with our work, these studies did not take into account the effect of WBC and TC on the hyperuricemia and MetS relationships when adjusting covariates. However, MetS and related insulin resistance are increasingly recognized as chronic low-grade inflammation (31, 32). A previous study has shown that the neutrophil to lymphocyte ratio was a valid bio-marker of MetS (33). Therefore, there may be confounding effects of LYMH in the relationship between uric acid and MetS. After adjustment for confounding factors, our results are stable (3); This discrepancy may be due to differences in lifestyle, and eating habits among the subjects of these studies.

### Hyperuricemia was positively correlated with the MetS components

The present study further showed that hyperuricemia was signifficantly associated with MetS-related variables such as elevated blood pressure, abdominal obesity, and elevated TG but not elevated fasting glucose and reduced HDL-C, which is slightly different from other studies. Many studies have demonstrated a strong association between SUA and various MetS-related components, but the association could be differed by study objects (25, 34, 35). Kim et al. discovered that hyperuricemia was positively connected with the remaining MetS components except for hyperglycemia in male patients, and hyperuricemia was favorably correlated with all MetS components in female patients (25). Feng et al. suggested that hyperuricemia was associated with elevated TG and elevated blood pressure, but not with elevated FPG and reduced HDL-C (34). In addition, Tian et al. found that all components of the MetS were positively associated with hyperuricemia (35). The inconsistencies in results between research could be due to the various populations studied and the various definitions of MetS utilized.

### Prevalence of MetS and its components

The unstandardized overall prevalence of MetS in our study was 17.3%, which was greater than the prior prevalence among farmers and herders in Lhasa (8.2%) and Tibetans on the Qinghai plateau (8.0%) (8, 36). It was considerably greater than the prevalence of MetS found in a 2002 epidemiological study (2.6%, after standardization for age and sex) (7). In the present study, no significant difference was observed in the prevalence of MetS between males and females. These results are consistent with the findings reported in Bangladesh (12, 17). In contrast, it was shown in Iranian research that more Iranian women (42%) than males (24%) had MetS (37). Women were also found to be a predictor of MetS in some previous studies (14, 18). Sex differences in the prevalence of MetS were thought to be due to cultural factors. However, in Saudi Arabia and Iran, the disparity could be due to differences in physical activity levels between males and females. A female is confined to the house for social and protective reasons, whereas a male is allowed to play or work outside with his friends and colleagues (18).

The prevalence of components such as abdominal obesity (46.3%) and reduced HDL-C (65.5%) was elevated significantly than elevated blood pressure (22.8%) and elevated fasting glucose (7.5%). These findings were similar to those from Bangladesh (12), but not to those from Lhasa (8). Fasting hyperglycemia (57.5%), abdominal obesity (46%), and hypertension (37%) were reported to be prevalent components of the MetS by Lhamo et al. (8). Farmers and herders with lower education made up the study population, and they observed that the prevalence of MetS was correlated with both low levels of education and insufficient physical exercise. They also found that the individuals had poor self-awareness, treatment, and management of their diabetes and dyslipidemia (8). Farmers and herders made up only 6.5% of our study population, and 73.9% of those with a secondary degree or higher. Hypertension and hyperglycemia may have been lowered as a result of increasing health knowledge, diet control, and physical activity. In the present study, the distribution of MetS components was uneven in Tibetan men and women, with abdominal obesity and reduced HDL-C predominating in women, and elevated triglycerides and blood pressure in men, which is inconsistent with studies from Bangladesh. As we can observe, the prevalence of the components of the MetS may be different among race groups, and genetic factors could be playing a role in this matter.

### Prevalence of hyperuricemia

In the present study, the mean concentration of serum uric acid was 374.8 ± 90.0 umol/L, hyperuricemia was more manifest in male subjects, which is similar to previous studies (12, 34). There were 125 (40.7%) subjects with hyperuricemia, much higher than the percentage in Bangladesh (16.6%) and the general Chinese population (8.4–25%) (10, 12). SUA levels are influenced by a variety of factors, such as diet, geography, sex, and genetics et al. (9, 38). The study in Bangladesh did not include alcohol users, but 55% of our individuals had previously consumed alcohol, while studies have shown that drinking alcohol increases SUA levels (39). The Tibetans preference for red meat may also contribute to elevated SUA levels, whereas the Bangladeshi diet is carbohydrate-based (12).

### Clinical value of this study

The clinical value of this study is as follows: (1) To our best knowledge, the independent association of hyperuricemia with MetS has not been developed in Tibetans on the Tibetan plateau, and our study found that hyperuricemia was significantly associated with MetS; (2) The findings of this study should be helpful for future research on the establishment of diagnostic or predictive models of metabolic syndrome in Tibetan populations.

### Strengths and limitations

Our study has some strengths: (1) This study is an observational study and therefore susceptible to potential confounding. We used strict statistical adjustment to minimize residual confounders. (2) We handled the target independent variable as both a continuous variable and a categorical variable. Such an approach can reduce the contingency in the data analysis and enhance the robustness of results; (3) Our multi-model adjustments during regression analysis showed that our results were stable; (4) We used another diagnostic criterion (NCEP-ATP III) to define MetS and showed stable results after multi-model adjustments; (5) Our study extends these findings by showing association of hyperuricemia with MetS in Tibetans on the Tibetan plateau.

This study has some limitations. First, although the sample size in the present study was relatively small, however, we performed multiple sensitivity analyses to ensure the robustness of the results. Second, according to our inclusion and exclusion criteria, we exclude Age<18 years, eGFR<60 mL/min/1.73m^2^, self-reported history of severe chronic illness, and pregnant and lactating women, therefore, the findings of this study cannot be used for these people. Third, we adjusted possible confounders to determine the relationship between SUA and MetS, but the influence of other unmeasured confounding factors could not be ruled out. For example, dietary intake can affect SUA levels, and physical activity can affect weight and blood pressure. But we have no information on the precise quantity of dietary intake and physical activity, future studies may consider collecting this information to explore the effects of dietary intake and physical activity on the relationship between hyperuricemia and MetS. Furthermore, these results are from a single center, and the interpretation of the finding of all Tibetans on the Tibetan Plateau may be limited. Finally, as a cross-sectional study design, it has less power to infer the causal relationship between serum uric acid and metabolic syndrome. Thus, additional prospective studies are needed to verify these findings in the future.

## Conclusions

In our study, hyperuricemia was significantly associated with the prevalence of MetS, and these relationships were not affected by sex or age. We also found that hyperuricemia was signiffcantly associated with MetS-related variables such as abdominal obesity, elevated blood pressure, and elevated triglycerides but not elevated fasting glucose and reduced HDL-C. Given the high prevalence of MetS and hyperuricemia among Tibetan adults, more studies are required to explore the role of SUA in the pathogenesis of MetS.

## Data availability statement

The raw data supporting the conclusions of this article will be made available by the authors, without undue reservation.

## Ethics statement

The studies involving human participants were reviewed and approved by The Hospital of Chengdu Office of People’s Government of Tibetan Autonomous Region ethics committee. The patients/participants provided their written informed consent to participate in this study.

## Author contributions

SY participated in the design of research schemes, collect and sort out data, and wrote the main manuscript text, and YZ, QZ, SB, HF, WG assist in data collection and LX participated in the design of research schemes. All authors contributed to the article and approved the submitted version.

## Funding

This study was supported by the hospital-level research project of the Hospital of Chengdu Office of People’s Government of Tibetan Autonomous Region Chengdu, China (grant number:2019-YJ-3).

## Acknowledgments

We thank Free Statistics team for providing technical assistance and valuable tools for data analysis and visualization. We thank Dr. Liu Jie (People’s Liberation Army of China General Hospital, Beijing, China) and Dr. Yang Qilin (The Second Affiliated Hospital of Guangzhou Medical University, Guangzhou, Guangdong, China) for helping in review and comments regarding the manuscript.

## Conflict of interest

The authors declare that the research was conducted in the absence of any commercial or financial relationships that could be construed as a potential conflict of interest.

## Publisher’s note

All claims expressed in this article are solely those of the authors and do not necessarily represent those of their affiliated organizations, or those of the publisher, the editors and the reviewers. Any product that may be evaluated in this article, or claim that may be made by its manufacturer, is not guaranteed or endorsed by the publisher.
