# Supplementary material for: Association between hyperuricemia and metabolic syndrome: A cross-sectional study in Tibetan adults on the Tibetan plateau
Source: Front Endocrinol (Lausanne). 2022 Oct 20;13:964872. doi: 10.3389/fendo.2022.964872 (PMC9632950; doi:10.3389/fendo.2022.964872)
Supplement: Supplementary file 1 [file DataSheet_1.docx]

Supplementary Material

# Supplementary Figures and Tables

## Supplementary Tables S1

| Table S1 Multivariable logistic regression analysis of the association between serum uric acid and metabolic syndrome (NCEP-ATP III). | | | | | | | | |
| --- | --- | --- | --- | --- | --- | --- | --- | --- |
| **Variables** | **Model1** | |  | **Model2** | |  | **Model3** | |
|  | **OR（95%CI）** | ***p* value** |  | **OR（95%CI）** | ***p* value** |  | **OR（95%CI）** | ***p* value** |
| UA per10 | 1.05 (1.01~1.1) | 0.008 |  | 1.12 (1.06~1.19) | <0.001 |  | 1.12 (1.06~1.19) | <0.001 |
| Hyperuricemia | 5.67 (2.47~13.01) | <0.001 |  | 6.42 (2.68~15.38) | <0.001 |  | 6.97 (2.79~17.41) | <0.001 |
| Model 1:no adjusted; | | | | | | | | |
| Model 2: adjusted for age, and sex; | | | | | | | | |
| Model 3: adjusted for age, sex, white blood cell, albumin, cholesterol; | | | | | | | | |
| Abbreviations: NCEP-ATP III, Adult Treatment Panel III of the National Cholesterol Education Program; UA, uric acid; CI, conﬁdence interval; OR, odds ratios; | | | | | | | | |

## Supplementary Tables S2

| Table S2 Multivariable logistic regression analysis of the association between serum uric acid and metabolic syndrome. | | | | | | | | | | | | | | | |
| --- | --- | --- | --- | --- | --- | --- | --- | --- | --- | --- | --- | --- | --- | --- | --- |
| **Variables** | **Model 1** | |  | **Model 2** | |  | **Model 3** | |  | **Model 4** | |  | **Model 5** | |  |
|  | **OR(95%CI)** | ***p* value** |  | **OR(95%CI)** | ***p* value** |  | **OR(95%CI)** | ***p* value** |  | **OR(95%CI)** | ***p* value** |  | **OR(95%CI)** | ***p* value** |  |
| **Uric acid**  **per10 umol/L** | 1.04 (1.01~1.08) | <0.001 |  | 1.09 (1.04~1.14) | <0.001 |  | 1.09 (1.04~1.14) | <0.001 |  | 1.09 (1.04~1.14) | <0.001 | | 1.09 (1.04~1.14) | <0.001 |  |
| **Subgroups** |  |  |  |  |  |  |  |  |  |  |  |  |  |  |  |
| Normouricemia | reference | |  | reference | |  | reference | |  | reference | |  | reference | |  |
| Hyperuricemia | 3.52 (1.88~6.57) | <0.001 |  | 3.87 (1.99~7.51) | <0.001 |  | 4.01 (2.02~7.99) | <0.001 |  | 4.03 (2.00~8.11) | <0.001 | | 4.02 (2.00~8.10) | <0.001 |  |
| Model 1:no adjusted; | | | | | | | | | | | | | | | |
| Model 2: adjusted for age, and sex; | | | | | | | | | | | | | | | |
| Model 3: adjusted for age, sex, white blood cell, albumin, and cholesterol; | | | | | | | | | | | | | | | |
| Model 4: adjusted for model 3 plus eGFR, Lymphocyte count, and alcohol consumption; | | | | | | | | | | | | | | | |
| Model 5: adjusted for model 4 plus Family history of Cardiovascular diseases, Family history of Hypertensive, Family history of Type 2 diabetes mellitus; | | | | | | | | | | | | | | | |
| Abbreviations: OR, odds ratios; CI, confidence interval. | | | | | | | | | | | | | | | |

## Supplementary Tables S3

| Table S3 Stratiﬁed analyses and interaction tests of the association between hyperuricemia and metabolic syndrome. | | | | | | |
| --- | --- | --- | --- | --- | --- | --- |
| **Subgroup** | **N event(%)** | **Crude OR (95CI)** | **Crude *p* value** | **Adj OR (95CI)** | **Adj *P* value** | ***P* for interaction** |
| **Male** |  |  |  |  |  | 0.603 |
| Normouricemia | 4 (4.7) | 1(Ref) |  | 1(Ref) |  |  |
| Hyperuricemia | 7 (15.2) | 3.68 (1.02~13.32) | 0.047 | 3.03 (0.57~16.1) | 0.193 |  |
| **Female** |  |  |  |  |  |  |
| Normouricemia | 14 (14.6) | 1(Ref) |  | 1(Ref) |  |  |
| Hyperuricemia | 28 (35.4) | 3.22 (1.55~6.68) | 0.002 | 3.73 (1.63~8.53) | 0.002 |  |
| **Age<40** |  |  |  |  |  | 0.148 |
| Normouricemia | 7 (10.1) | 1(Ref) |  | 1(Ref) |  |  |
| Hyperuricemia | 15 (18.8) | 2.04 (0.78~5.35) | 0.145 | 2.06 (0.64~6.66) | 0.225 |  |
| **Age>=40** |  |  |  |  |  |  |
| Normouricemia | 11 (9.7) | 1(Ref) |  | 1(Ref) |  |  |
| Hyperuricemia | 20 (44.4) | 7.42 (3.15~17.46) | <0.001 | 4.52 (1.7~12.03) | 0.002 |  |
| **Altitude<3700** | |  |  |  |  | 0.241 |
| Normouricemia | 11 (11.6) | 1(Ref) |  | 1(Ref) |  |  |
| Hyperuricemia | 15 (25.4) | 2.6 (1.1~6.15) | 0.029 | 2.34 (0.84~6.47) | 0.103 |  |
| **Altitude>=3700** | |  |  |  |  |  |
| Normouricemia | 7 (8) | 1(Ref) |  | 1(Ref) |  |  |
| Hyperuricemia | 20 (30.3) | 4.97 (1.95~12.64) | 0.001 | 5.85 (1.94~17.62) | 0.002 |  |
| Adjusted for age, sex, cholesterol, eGFR, WBC, LYMPH, ALT, AST, Alcohol consumption, albumin, Family history of CVD, family history of hypertensive, family history of T_2_DM; | | | | | | |
| Abbreviations: OR, odds ratios; CI, confidence interval; Adj, adjusted; Ref, reference; | | | | | | |
